# Applications of proteochemometrics - from species extrapolation to cell line sensitivity modelling

**DOI:** 10.1186/1471-2105-16-S3-A4

**Published:** 2015-02-13

**Authors:** Isidro Cortes-Ciriano, Gerard JP van Westen, Daniel S Murrell, Eelke B Lenselink, Andreas Bender, Therese E Malliavin

**Affiliations:** 1 Institut Pasteur, Unité de Bioinformatique Structurale; CNRS UMR 3825; Département de Biologie Structurale et Chimie, 25, rue du Dr Roux, 75015, Paris, France; 2 ChEMBL Group, European Molecular Biology Laboratory European Bioinformatics Institute, Wellcome Trust Genome Campus, CB10 1SD, Hinxton, Cambridge, UK; 3 Centre for Molecular Science Informatics, Department of Chemistry, University of Cambridge, Cambridge, UK; 4 Division of Medicinal Chemistry, Leiden Academic Center for Drug Research, Leiden, The Netherlands

## Background

Proteochemometrics (PCM) is a predictive bioactivity modelling method which simultaneously models the bioactivity of multiple ligands against multiple targets. PCM permits exploration of the selectivity and promiscuity of ligands on biomolecular systems of different complexity. This includes proteins and even cell-line models [1,2]. The suitability of PCM to predict compound polypharmacology has been validated both retrospectively and in prospective experimental validation [1,2]. In practice, each ligand-target interaction is encoded by the concatenation of ligand and target descriptor vectors used to train a single machine learning model. The inclusion of both chemical and target information enables the extra- and interpolation on the chemical and on the biological space. Therefore, PCM permits to predict compound bioactivities on targets not present in the training phase [3].

## Results

In this contribution, we show a methodological advancement in the field [4], namely how Bayesian inference (Gaussian Processes) can be successfully applied in the context of PCM for (i) the prediction of compound bioactivity along with the error estimation of the prediction; (ii) the determination of the applicability domain of a PCM model; and (iii) the inclusion of experimental uncertainty of bioactivity measurements. We illustrate how the application of PCM can be useful in medicinal chemistry to concomitantly optimize compounds selectivity and potency, in the context of two application scenarios: (a) modelling isoform-selective cyclooxygenase inhibition; and (b) large-scale cancer cell line drug sensitivity prediction, where we benchmark the predictive signal of basal gene expression, gene copy-number variation, exome sequencing, and protein abundance data. We present the R package **C**hemically **A**ware **M**odel **B**uilder (*camb*) [5], which is able to perform the above mentioned modelling tasks. *camb *is an open source platform for the generation of Structure-Activity and Structure-Property models. The functionalities of *camb *include: (i) standardisation of chemical structure representation, (ii) calculation of 905 one-dimensional descriptors and 14 fingerprints for small molecules, (iii) 8 types of amino acid descriptors, (iv) 13 whole protein sequence descriptors, and (iv) training, validation and visualization of predictive models.

## Conclusions

Overall, the application of PCM in these two case scenarios let us conclude that PCM is a suitable technique, on this data, to model the activity of ligands exhibiting diverse bioactivity profiles across a panel of targets, which can range from protein binding sites (a), to cancer cell-lines (b). The *camb *package constitutes a platform encompassing all steps for the generation of predictive models from chemical structures and their associated bioactivities/properties, which will provide reproducibility and simplify the generation of predictive bioactivity/property models.
